# Promoting Inclusivity in Dementia Education and Screening in an Ethnically Diverse, Rural Community During a Pandemic

**DOI:** 10.1093/geroni/igab046.125

**Published:** 2021-12-17

**Authors:** Lisa Wiese, Ishan Williams, Nancy Schoenberg, James Galvin, Jennifer Lingler

**Affiliations:** 1 Florida Atlantic University, C.E. Lynn College of Nursing, Florida, United States; 2 University of Virginia, Charlottesville, Virginia, United States; 3 University of Kentucky, Lexington, Kentucky, United States; 4 University of Miami Miller School of Medicine, Miami, Florida, United States; 5 University of Pittsburgh, Pittsburgh, Virginia, United States

## Abstract

Rural, ethnically diverse older adults experience disparities in dementia detection/management. The Covid-19 quarantine exacerbated these disparities, and threatened faith-based dementia education and screening activities. We investigated the effectiveness of a telephone-based outreach for increasing dementia knowledge and detecting cognitive risk among a rural, diverse, underserved community of 89% African American, Hispanic, and Haitian Creole residents, Faith-based health educators, trained using virtual Alzheimer’s Association resources, contacted church congregants who responded to radio worship service announcements. Participants completed telephone measures of basic dementia knowledge and cognitive risk. Of the estimated 120 persons across five churches who received an invitation, 75% (n = 90) participated in dementia education and memory screening via telephone. Twelve (80%) of the 15 participants assessed as being at risk followed up with their provider. Rural residents are known for preferring face-to-face contact. Their willingness to complete health-promoting research activities by telephone highlighted the community’s interest in dementia awareness.

